# Robot-assisted deep brain stimulation of the centromedian nucleus of the thalamus for generalized epilepsy: targeting and operative video

**DOI:** 10.3171/2024.4.FOCVID245

**Published:** 2024-07-01

**Authors:** Jason A. Chen, Aaron E. L. Warren, John D. Rolston

**Affiliations:** Department of Neurosurgery, Brigham and Women’s Hospital, Boston, Massachusetts

**Keywords:** deep brain stimulation, centromedian nucleus, thalamus, generalized epilepsy

## Abstract

The centromedian (CM) nucleus of the thalamus is a promising target for a range of brain diseases including drug-resistant generalized and multifocal epilepsy. CM is highly connected to cortical and subcortical regions including frontoparietal/sensorimotor cortex, striatum, brainstem, and cerebellum, which are involved in some generalized epilepsy syndromes like Lennox-Gastaut syndrome (LGS). In this video, the authors describe their methodology for targeting CM for deep brain stimulation (DBS). Delineation of an optimal and consistent target will expand the efficacy of neuromodulation of CM in intractable epilepsy.

The video can be found here: https://stream.cadmore.media/r10.3171/2024.4.FOCVID245

## Transcript

### 0:21 Introduction.

Here we review our methodology for targeting the centromedian (CM) nucleus and surgical procedure for CM deep brain stimulation (DBS) in a patient with intractable generalized epilepsy. CM has high connectivity to cortical and subcortical regions including sensorimotor cortex, striatum, brainstem, and cerebellum. Efficacy data supporting CM-DBS, including the 2022 ESTEL trial in patients with Lennox-Gastaut syndrome (LGS),^1,2^ reveal the importance of accurate target selection.

### 0:48 Patient History.

The patient is a 33-year-old man with medically intractable epilepsy and intellectual disability with seizure onset occurring at 2 years of age. Video-EEG confirmed electroclinical features consistent with the diagnosis of LGS, including multifocal seizure onset with bilateral tonic arm flexion, disorganized EEG background with diffuse polymorphic delta activity, and intermittent generalized paroxysmal fast activity (GPFA). He had other seizure types including bilateral tonic-clonic seizures, focal seizures, and a recent episode of nonconvulsive status epilepticus. Seizures did not respond to insertion of a vagal nerve stimulation (VNS) device at age 24. We planned for CM-DBS following a multidisciplinary epilepsy conference.

### 1:35 Targeting.

CM is difficult to delineate on standard MRI sequences.^3^ We obtain a white matter–nulled MPRAGE sequence in preoperative imaging.^4^ Then, the Thalamus Optimized Multi-Atlas Segmentation (THOMAS) method is used to segment the thalamus into individual nuclei.^5^ Here the CM is labeled in yellow. The CM and the so-called "sweet spot" defined from the ESTEL investigators^6^ are burned into the preoperative scans for targeting during surgery. We plan a trajectory to a point between this sweet spot and the canonical CM.

### 2:16 Surgical Procedure.

Surgery is performed under general anesthesia (0.15 μg/kg/min remifentanil and 120 μg/kg/min propofol). We affix a Leksell frame to the patient’s head. A CT scan is obtained with the O-arm, and this new scan is aligned to the preoperative imaging. The patient’s head is then affixed to the ROSA robot and secured. At this point we unplug the bed to prevent movement of the body, as the ROSA holds the head. ROSA is a robotic stereotactic assistance system that relies on fusion of the obtained O-arm scan to the preoperative imaging, including the target, and registration of the scan with fiducial landmarks (here, the pins of the frame), to define the stereotactic space. We make incisions of approximately 3 cm at each of the planned entry points. With the assistance of the ROSA robot, a 3.2-mm burr hole is created at each of these entry points.

### 3:27 Microelectrode Recording.

The ROSA arm was driven to 160 mm above the target, in preparation for microelectrode recording. The Alpha Omega microdrive attaches to the ROSA arm, and a guide cannula is inserted through the center channel of the Ben-Gun. At this point we ensure the blood pressure is low to avoid the risk of hemorrhage.

The microelectrode is threaded into the cannula, and advanced along the planned trajectory, starting 15 mm superficial to the target. Here is a sample of the microelectrode recording from this case.

At 5 mm above target, which was the expected entry into CM, we observed a reduction in spiking and background activity, corroborating our trajectory’s accuracy and helping determine the final electrode depth. We have seen this reduction in activity in all CM cases thus far, and it is consistent with the reduced neuronal density of this structure.^3,7^

### 5:03 DBS Lead Placement.

The electrode is placed onto the robot arm and into the brain at the targeted depth. The stylet is removed, and the guide cannula is carefully extracted. On the left side, the electrode is tunneled to the right side and secured in place with a dog-bone plate. The process of microelectrode recording and electrode placement is repeated for the contralateral side. We then carefully tuck the leads into a subgaleal pocket. The surgical sites are irrigated with saline and instilled with vancomycin powder. The O-arm confirms placement of the electrodes, visualized with the planning software.

### 6:00 IPG and Lead Extender Placement.

Next, we detach the patient from the robot and reposition him for implantation of the pulse generator (IPG). In the chest, we dissect a pocket in the subfascial plane; from the scalp incision, a tunneler is passed to the chest incision. We attach the lead extenders, bring the leads through the tunnel, and connect it to the pulse generator. Impedances are reassuring. The scalp and chest incisions are closed.

### 6:27 Postoperative Course.

We obtain a postoperative CT scan (0.5-mm slice thickness). Using the Lead-DBS software,^8^ we verify the lead positions. Here, we placed the electrode between the CM and the ESTEL "sweet spot," as initially planned. Programming settings followed the ESTEL and SANTE^9^ trials, with pulse width of 90 microseconds and frequency of 145 Hz. Future follow-up will assess the degree of symptomatic improvement.

## Acknowledgments

We acknowledge the support of our operating room staff in helping to create this video; the BWH Epilepsy Team; and dedicate our work to our patients and their families.

## Disclosures

Dr. Chen reports equity in Verge Genomics and Gravity Medical Technology.

## Author Contributions

Primary surgeon: Rolston. Assistant surgeon: Chen. Editing and drafting the video and abstract: all authors. Critically revising the work: all authors. Reviewed submitted version of the work: all authors. Approved the final version of the work on behalf of all authors: Rolston. Supervision: Rolston.

## Supplemental Information

### Patient Informed Consent

The necessary patient informed consent was obtained in this study.
